# The Glories of the Profession and Non-Combatants

**Published:** 1919-03-29

**Authors:** 


### The Hospital

The Workers' Newspaper of Administrative Medicine and Institutional Life, Administration, National Insurance and Health.

No. 1712, Vol. LXV. SATURDAY, MARCH 29, 1919. PRICE TWOPENCE

### THE GLORIES OF THE PROFESSION AND NON-COMBATANTS.

We have often written of the claims which the higher placed hospital workers especially, who have devoted themselves throughout the war to the hospitals and their patients in the Homeland, have on the nation and Empire. They have displayed splendid devotion to provide from the outset that every administrative detail, and all that appertains to the highest efficiency in treatment and results, should be continuously forthcoming everywhere throughout the Homeland for our wounded and sick warriors of all the Services. It has been a new departure, and a welcome one, for those responsible for recognition by the bestowal of honours and otherwise in the present, which is the greatest war in the world's history, that service and devotion can be marked by special signs, indicating the fields in which distinction has been won. We feel that there should be established a special distinction attached exclusively to Homeland Service of the highest type. From His Majesty the King downwards, irrespective of age, and wholly regardless of the heavy strain on individuals which has resulted from the zealous and continuous war work undertaken, courage, devotion and vital service of the highest type have been nowhere manifested to a greater extent than by many of these responsible Homeland workers.

Members of the medical profession of nearly all grades have made great sacrifices, and risked much throughout the war in their work for the nation. The necessities of war time and war changes have revolutionised in many cases the incidence and character of hospital work at home, which has entailed upon members of the medical profession longer hours on duty and more and more work, without adequate compensation or recognition. At times, when the fighting has been hardest, and the battles prolonged, the relays of wounded who have continuously been sent home for hospital care and treatment have kept members of the staff of all grades continuously employed, and surgeons have never been sure of a quiet night's rest, nor could they arrange what any day would require at their hands, nor know the hour of the day or night when their services would be claimed. Without the slightest hesitation these noble workers have put aside everything else, that they might devote themselves to the work which the urgent needs of the wounded demanded at their hands.

The services of the profession at the front, day in and day out for many days and nights continuously, have been one of the most glorious chapters in the history of British and American medicine. No one who has not been familiar with the outposts, receiving stations, and all the many and varied types of hospitals and accommodation created during the war can realise, even faintly, what the strain has been, nor the voluntary and glorious output of the workers. To an extent workers at the front, or the chief of them, may have been granted higher remuneration and recognition, while the sailors and soldiers have recognised and testified to the splendid work of the profession at the front. It has placed the whole nation and Empire, and all the people of the Allied countries, under continuous obligation to physicians and surgeons, and to the younger members of the profession, who have so gloriously given everything they had of the best to the work of meeting the special and ever-varying demands, arising out of the latest inhuman methods of conducting the war. It seems to us that the time has arrived when testimony should be borne to the claims and valour of the non-fighting services and all who have laboured to keep them at such a standard of excellence, as to make the devil's methods of war as little harmful and permanently disabling, as has been humanly possible, to our Warriors of all ranks.

Those members of the profession whose field of work has been mainly or entirely in the Homeland have had many disabilities and ever-increasing and more arduous claims on their time and services than the most experienced of them could have believed possible. Some of the best and bravest, the strongest, aye, and the oldest of the Homeland workers, who had attained high rank and positions of leadership before the war, have found, as the war proceeded, that the pressure and claims upon them have been so heavy at times as to break down the strength of the worker, and add thereby to the difficulty of continuing the work as others fell out, and the number of hospitals and patients increased. Some others may have increased their earnings. But how many consultants have found their practice disappearing, while their hours of work have continuously increased, because very many of their private patients before the war, when wounded and requiring operation and other treatment, became patients at a war hospital to which the consultants were giving their services freely and wholeheartedly?

It is right that the people of these islands, and the people of the nations of the Allies, should have these facts brought to their notice, that they may be placed in a position to realise the great work so devotedly done for their nearest and dearest, who have fought so valiantly on the battlefields, and to whose valour we owe the glorious victory that in God's blessing has attended our Navy and Armies. The pressure has been so great, the work so overpowering and pressing, and the ever-changing and varying circumstances which have distracted public attention so constant, as to make it well-nigh impossible for even the wisest and most alert of nations to be in a position, as yet, to weigh individual achievements, or to become aware of the magnitude of the services which have been rendered throughout the war. The time has come to provide for the organisation of a great national welcome and thanksgiving to the non-fighting and scientific sections of war workers. Their magnificent and successful services will go down to history as having contributed to the saving of human life, and the reduction of mortality in the field, to an extent, which has made the splendour of these hygienic and preventive measures wonderful to scientists, and should be brought home too, to the people at large.

